# Molecular evidence for relapse of an imported *Plasmodium ovale wallikeri* infection

**DOI:** 10.1186/s12936-018-2226-4

**Published:** 2018-02-09

**Authors:** Luzia Veletzky, Mirjam Groger, Heimo Lagler, Julia Walochnik, Herbert Auer, Hans-Peter Fuehrer, Michael Ramharter

**Affiliations:** 1 Division of Infectious Diseases and Tropical Medicine, Department of Medicine I, Medical University of Vienna, Vienna, Austria; 2 Institut für Tropenmedizin, Universität Tübingen, Tübingen, Germany; 3 Institute for Specific Prophylaxis and Tropical Medicine, Medical University of Vienna, Vienna, Austria; 4 Department of Pathobiology, Institute of Parasitology, University of Veterinary Medicine Vienna, Vienna, Austria; 5 Bernhard Nocht Hospital for Tropical Diseases, Bernhard Nocht Institute for Tropical Medicine and University Medical Center Hamburg-Eppendorf, Hamburg, Germany

**Keywords:** Malaria, *Plasmodium ovale*, *Plasmodium ovale wallikeri*, Relapse, Case report

## Abstract

**Background:**

Malaria caused by *Plasmodium ovale* spp. has been neglected by and large from research and has received only little scientific attention during the past decades. Ovale malaria is considered to feature relapses by liver hypnozoites although scientific evidence for this paradigm is scarce.

**Case presentation:**

Here, the case of a 16-year-old male, who presented with fevers to the outpatient department in Vienna, Austria, after travelling to Uganda and Papua New Guinea is described. Infection with *Plasmodium malariae* was diagnosed by microscopy and the patient was treated accordingly with a full course of supervised artemether–lumefantrine. He was discharged in good clinical condition with a negative blood smear. One month after initial diagnosis, he returned complaining of fever. Thick blood smear was positive again for malaria parasites, which were confirmed as *P. ovale wallikeri* by PCR. Retrospective analysis revealed the identical *Plasmodium* spp. in the initial blood samples. Molecular analysis of various gene loci (*nuclear porbp2*, *18S rRNA* and *potra* genes) gave identical results providing further evidence for relapse by an identical parasite genotype. Consecutively, the patient was retreated with artemether–lumefantrine and received a regimen of primaquine according to WHO guidelines.

**Conclusion:**

Conclusive evidence for relapses with *P. ovale* spp. is rare. The presented case provides convincing confirmation for the relapse paradigm based on re-appearing parasitaemia following supervised treatment in a non-endemic region with a parasite strain of identical genotype.

## Background

*Plasmodium ovale* malaria has been neglected largely from research in the past decades with the primary focus being laid on falciparum and to a lesser extent vivax malaria. Even though evidence is scarce, *P. ovale* is considered a relapsing *Plasmodium* spp. by analogy to *Plasmodium vivax* for which evidence for liver hypnozoites is well-established [1].

In general, a recurrence of parasites after initial treatment can be due to recrudescence of a not completely cleared bloodstream infection, to a new infection or to a relapse originating from dormant liver stages. The existence of *P. ovale* hypnozoites was, however, never firmly established nor has a clinical trial on the nature of such relapses been performed [2]. Most of the information available on *P. ovale* relapses has derived from observations from malaria infection therapy for neurosyphilis in the first half of the 20th century or from case reports. Further evidence making use of molecular methods is largely missing. Astonishingly, as recently described by a systematic review, from the first description of the species in 1922 until 2015 less than 20 case reports have been published on potential relapses of *P. ovale* spp. [2]. Further evidence proving or refuting the relapse paradigm for *P. ovale* is, therefore, warranted not least to provide evidence for current guidelines for anti-hypnozoitocidal treatment with primaquine.

## Case presentation

A 16-year-old male patient presented with a 5-day history of recurrent fever attacks up to 39.5 °C and with night sweats and arthralgia to the outpatient department (OPD) of the infectious diseases ward of the General Hospital of Vienna. Laboratory examination indicated a moderately increased CRP (1.57 mg/dL) with a normal white blood count, thrombocytopaenia of 105 g/L, a moderately raised total bilirubin of 2.33 mg/dL in addition to moderately increased LDH of 277 U/L. Differential blood count showed monocytosis and atypical lymphocytes. A rapid diagnostic influenza test (Xpert^®^ Flu, Cepheid) was negative for Influenza A and Influenza B, as well as ELISAs for CMV and EBV. An aerobic blood culture was positive for *Staphylococcus hominis* after 20.4 h. This was not confirmed by other simultaneously drawn blood culture sets and was thus regarded as contamination. Physical examination was normal apart from an enlarged sub-angular lymph node on the right side. The anamnesis revealed that the patient had returned from a trip of 1 ½ months in duration, to Uganda, Papua New Guinea and Singapore, 3 months before the onset of symptoms. During this stay and until 3 weeks after returning home, he took continuous malaria prophylaxis consisting of mefloquine (Lariam^®^) once per week. He did not report any associated complaints. Eight months before, he had stayed at the Philippines for 10 days.

Thick and thin blood smears were performed and *Plasmodium malariae* was diagnosed microscopically. A routine therapy with six adult doses of Riamet^®^ (artemether–lumefantrine) was initiated according to the manufacturer’s recommendations and following WHO treatment guidelines. On the first day of treatment body temperature increased to a maximum of 39.8 °C, but the general health status improved subsequently. A thick blood smear on the last day of treatment was negative and the patient was discharged in good condition. A follow-up examination was planned for 1 week after admission to which the patient, however, did not present.

One month later, the patient returned to the outpatient department with reported fever. He had not travelled abroad since the initial malaria diagnosis. A thick blood smear showed a recurrence of parasites with a very low parasitaemia. The patient was retreated with Riamet^®^ and samples from the first and second fever episode were sent for species specific PCR diagnosis [3]. PCR from EDTA blood detected a *P. ovale wallikeri* mono-infection at both time points. Further analyses were initiated with the aim to identify whether the second attack was a relapse of the same parasite isolate or a prolonged latency of another *P. ovale wallikeri* parasite obtained during the preceding trips.

Molecular analysis of several gene loci was conducted to evaluate both isolates. Initially, a fragment of the nuclear *18S rRNA* gene was analysed to identify *Plasmodium* spp. (Primers rOVA1WC/rOVA2WC) [4]. The positive isolates were further analysed for discrimination between *P. ovale curtisi* (rOVA1/rOVA2) [5] and *P. ovale wallikeri* (rOVA1v/rOVA2v) [3]. To examine if the isolates were identical genotypes the nuclear *porbp2* and *potra* genes were analysed.

Examined sequences of the SSU rRNA, *porbp2* [6, 7] and *potra* [8] genes revealed homology of the two isolates. The sequences can be accessed in GenBank with the numbers (MG251661—*P. ovale wallikeri* isolate 17T1554/17T186R. porbp2, MG255222–MG255223—*P. ovale wallikeri* isolate 17T1554/17T186R SSU rRNA gene, partial sequence).

Accordingly, the patient’s glucose-6-phosphate dehydrogenase (G6PD) status was tested and showed normal enzyme function so that a 14 days course of primaquine was initiated [9]. During the following 7 months, the patient did not experience further episodes of disease.

## Discussion and conclusions

Detection of *Plasmodium* by microscopy of blood smears is still the gold standard for malaria diagnosis and provides a fast and valid diagnostic tool when performed by experienced slide readers. Since the introduction of species-specific diagnosis using PCR it has, however, become more and more evident that a considerable number of microscopically retrieved results are inaccurate, especially concerning species distinction in non-falciparum and mixed *Plasmodium* infections [10–12]. The here described case underlines the usefulness of diagnostic confirmation by PCR and its possible clinical importance.

This case adds valuable information to the scarce evidence available in the literature on the relapse hypothesis for *P. ovale* malaria. To current knowledge, no other case with subsequent molecular analysis of primary and recurring infection has been reported from a non-endemic area [2, 13]. Here, a re-infection can be excluded as the patient remained in a non-malaria endemic country after treatment of the initial malaria episode. Also, the possibility of prolonged latency of a different *P. ovale* parasite originally acquired at the same time causing the second episode is negligible as the obtained sequence data strongly suggest the presence of the identical malaria parasite during the initial and the recurring episodes. Here, sample amount was not sufficient but in upcoming studies whole genome sequencing is recommended to prove identical genotypes. More difficult is a distinction between recrudescence and relapse. However, the blood-stage medication was taken under observation and there are no known specific drug resistance mechanisms for *P. ovale* spp. against artemether or lumefantrine, thus a recrudescence seems very unlikely.

Anti-hypnozoite treatment with 8-aminoquinolines was introduced based on the observation that quinine therapy was more efficacious in combination with the 8-aminoquinoline pamaquine for the treatment of relapsing *P. vivax* infections than as monotherapy. Based on this, 8-aminoquinolines were further developed, but their effectiveness against relapsing *P. ovale* infections was merely presumed [14]. Models for testing hypnozoitocidal drugs for any species are not easy to develop which complicates research and development of new compounds that are also suitable for broad use. Recent experiments in humanized mice suggest the formation of *P. ovale* dormancies in human liver cells, if only for a limited time, and raise hope that more light will be shed on the relapse phenomenon [15, 16].

In 2015, Robinson et al. conducted a randomized double-blind placebo-controlled trial comparing blood stage drugs plus primaquine to blood stage drugs plus placebo in Papua New Guinean children [17]. The included patients were allocated to the respective treatment groups and followed for 32 weeks. Within this period, the qPCR-corrected time to the first clinical malaria episode was studied. The risk of having at least one PCR detectable *P. ovale* infection within the 8 months of follow up was significantly lower in the group that received primaquine. The same was true for *P. vivax*. For *Plasmodium falciparum* or *P. malariae*, however, no significant association of this kind could be observed. These results support the hypothesis of a quiescent parasite stage that is affected by primaquine. Yet it is to note that this was a limited sample size and no analyses were made regarding whether the observed episodes were following a previous *P. ovale* infection or not.

Interestingly, recent studies doubt the exclusive connection between liver dormancies and tertian malaria and suggest other havens of dormancies and a relapse potential of all human infective *Plasmodium* spp., respectively [1, 18, 19]. Indeed, when having a closer look at the history of publications on dormancies derived from non-human *Plasmodium* spp., dormant stages were also reported in the epidermis, brain, spleen, kidneys and lungs [20, 21]. Further case reports or small retrospective analyses of possible relapse cases can be found in the scientific literature, however, without supporting molecular analyses [22, 23].

This case report provides an important piece in the puzzle to substantiate the relapsing properties of *P. ovale*. Still, more evaluations of this kind are needed and the causal relationship between relapse and liver dormancies remains to be further explored.

## Abbreviations

ACT: artemisinin based combination therapy
CMV: cytomegalovirus
EBV: Epstein–Barr virus
G6PD: glucose-6-phosphate dehydrogenase
OPD: outpatient department
PCR: polymerase chain reaction
porbp2: *Plasmodium ovale* reticulocyte binding protein 2
potra: *Plasmodium ovale* tryptophan-rich antigen
spp.: species
SSU: rRNA small subunit ribosomal ribonucleic acid
WHO: World Health Organization
